# Reply to Rubin, J.M.; Kripfgans, O.D. Comment on “Barbieri et al. Umbilical Vein Blood Flow in Uncomplicated Pregnancies: Systematic Review of Available Reference Charts and Comparison with a New Cohort. *J. Clin. Med.* 2023, *12*, 3132”

**DOI:** 10.3390/jcm13092654

**Published:** 2024-04-30

**Authors:** Moira Barbieri, Enrico Mario Ferrazzi, Tamara Stampalija

**Affiliations:** 1 Department of Mother, Child and Neonate, Fondazione IRCCS Ca’ Granda Ospedale Policlinico di Milano, 20100 Milan, Italy; moira.barbieri@unimi.it (M.B.); enrico.ferrazzi@unimi.it (E.M.F.); 2 Department of Clinical and Community Sciences, University of Milan, 20100 Milan, Italy; 3 Department of Mother and Neonate, Institute for Maternal and Child Health IRCCS “Burlo Garofolo”, 34100 Trieste, Italy; 4 Department of Medicine, Surgery and Health Sciences, University of Trieste, 34100 Trieste, Italy

We thank the authors for the interest in our paper [1]. Dr. Rubin and Dr. Kripfgans [2] describe the umbilical vein blood flow (UV-Q) measurements by means of 2D imaging and the Doppler velocity interrogation as both “cumbersome and inaccurate” and propose their methodology that is assumed to be angle-, flow-profile- and vessel-geometry-independent.

First, let us agree on the importance of UV-Q measurement as a biological parameter that reflects the true fetal blood supply and its possible clinical applications. Regarding the criticisms that the authors raised for the described methodology of UV-Q measurement, we would like to address some comments.

**Parabolic flow.** The ideal model of blood flow is a parabolic flow uniformly distributed along the vessel. In this condition, the mean velocity can be calculated by using the following formula:

mean velocity=peak velocity×0.5

where 0.5 represents a correction coefficient derived by an ideal parabolic flow.

Close to the placenta, the flow profile is similar to a flat profile, in which the mean and peak velocities differ by a spatial velocity distribution coefficient of 0.7, whereas along the free loops, this coefficient is 0.6 [3]. Thus, the indication to sample a free loop derives from the observation that the velocity profiles are approximately flat at the placental insertion and become more parabolic in the free loop [3]. These flow velocity profiles may also modify along the cord, due to its curvature causing minor turbulences, and are not completely corrigible by the angle correction or by a simple coefficient. This complex hemodynamics should require a case-by-case correction. For these reasons, and in the absence of other validated techniques of UV-Q measurement, the 0.5 coefficient has been adopted by many authors as a simple coefficient that can be adopted for UV-Q assessment. It is not the first time that a systematic error has been accepted in medicine. For example, different techniques for the assessment of maternal hemodynamics parameters give different absolute values (i.e., cardiac output) [4,5]. Despite the lack of rigorous standardization and systematic differences between techniques, both the evaluation of the trend of these parameters and the observed measurements versus the reference values obtained with the same criteria are considered to be valuable in obstetric practice, although the techniques cannot be applied interchangeably. 

**Velocity blood flow measurement.** The mean or modal spatial velocity of the Doppler interrogation of the UV-Q could be calculated as the intensity-weighted modal velocity (IWMV) directly by pulsed Doppler equipment, but this value is generally highly influenced by noise produced by the neighboring vessels and wall [6,7] and might be additionally worsened by the effects of the high-pass filters. 

Although further investigations are required to examine the velocity profile through the umbilical cord, the measurement errors yielded by the maximum velocity method seem to be more predictable and systematic [8]. This is why we adopted the maximum velocity profile corrected by the 0.5 coefficient as per original experimental measurements on ovine fetal lamb [7,9].

**Vessel lumen measurement.** As regards the precision of the diameter measurement, we agree that the diameter derived from the cross-sectional area (CSA) of the vessel, when obtained from a 3D reconstruction, is prone to minor angle errors away from the exact perpendicular section, and this might introduce up to 20% of the mistakes, since the CSA is obtained by using the following formula:

CSA=π×(diameter/2)^2

where any error is squared and is as such amplified. However, the measurement of the diameter between the brightest echoes (inner to inner) in the vein wall constitutes a strong candidate for standard methodological recommendations, as validation studies in animal models have demonstrated the accurate quantification of UV-Q measurements by estimating the CSA through the inner diameter measurements on free-floating portions of the umbilical cord [7,10]. 

The inner diameter of the vessel is better obtained on a straight segment of the vein, as the average of multiple measurements [11]. In agreement with most of the authors who investigated UV-Q, we adopted “the front of back of the echo from the near wall to the front of the echo from the far wall” method. The brightest reflected echoes of the walls indicate the best perpendicular section on the largest diameter of the vessel. 

**Inaccuracy and reproducibility.** Adherence to the same methodology results in reasonably accurate measurements of UV-Q, as shown by us and others [2,8], which are comparable to values reported for the maternal and fetal Doppler velocimetry [12] and which are widely used in clinical settings despite a considerable heterogeneity in the reported reference ranges [13,14]. 

**New approaches to UV-Q quantification.** Traditional methods of UV-Q estimation rely on vessel diameter measurements from 2D B-mode ultrasound images and spectral Doppler for mean velocity estimates, but these are prone to an error quota, as discussed above. To address these limitations, Rubin and colleagues recently developed a new method for estimating blood volume flow that uses a mechanical 3D probe to measure the total integrated flux through an ultrasound-generated Gaussian surface that intersects the umbilical cord [2,15].

Even if this method seems to overcome several technical limitations of standard flow methods (as it is described by authors to be angle-independent, flow-profile-independent and vessel-geometry-independent), there are some limitations that should be noted. First, fetal motion becomes especially important to consider, as a time of approximately 8 mininutes was required to obtain enough samples for a statistically valid flow estimation [15]. Second, the size of the recruited population was limited. Furthermore, the biggest obstacle is that this method has not been implemented on any clinical ultrasound machine, making it impossible to use this new method.

In conclusion, we agree that there are few critical aspects of UV-Q quantification. However, these can be easily overcome by practice and by the adoption of a standardized technique. In support of this statement, the 2D–Doppler measurement of UV-Q has been found to be accurate when compared with several gold standards for in vivo flow calculation [7,16,17]. Particularly, UV-Q measurement by the 2D–Doppler methodology versus dilutional methodologies and microspheres achieved very good results (5.5% and 5.3% mean differences) [7,17].

As regards the cumbersome methodology, we should consider the enormous improvements in ultrasound equipment since the first studies on UV-Q [16,18]. Moreover, the 2D–Doppler methodology described usually takes no longer than 3–4 min for three repeated measurements. 

We indeed appreciate and compliment Dr Rubin and Dr Kripfgans for the possible technological and software innovation they have been working on since 2006, hoping it might be implemented in ultrasound equipment and in clinical settings once its accuracy has been tested through validation studies. In our opinion, until a proven alternative methodology or ultrasound software does not take its place, the 2D–Doppler methodology remains a valuable tool in maternal–fetal medicine to be adopted and promoted. 
